# Acute Effects of Esketamine on Blood Pressure and Heart Rate: Associations with Psychopathological Traits by Minnesota Multiphasic Personality Inventory 2 (MMPI-2)

**DOI:** 10.1192/j.eurpsy.2026.11794

**Published:** 2026-07-31

**Authors:** C. Serritella, L. Iorio, F. Ferraiuolo, L. Catalano, R. De Biase, D. Riccio

**Affiliations:** 1 UOSM 12; 2 Psicologia, Università Luigi Vanvitelli, Caserta; 3 Linguistica, Università telematica Pegaso, Milano, Italy

## Abstract

**Introduction:**

Intranasal esketamine provides a rapid therapeutic option for treatment-resistant depression but can induce transient blood pressure increases. The influence of psychopathological traits on these cardiovascular responses remains largely unexplored.

**Objectives:**

The present study aims to investigate the acute effects of intranasal esketamine on systolic and diastolic blood pressure as well as heart rate, and to examine their associations with psychopathological profiles as measured by the MMPI-2, including Harris and Lingoes subscales. Specifically, the study seeks to determine whether distinct psychopathological traits predict differential cardiovascular responses to esketamine administration.

**Methods:**

Ten patients receiving esketamine treatment were assessed at the Mental Health Centre of Caserta (UOSM-12). Systolic blood pressure (SBP), diastolic blood pressure (DBP), and heart rate (HR, beats per minute) were measured immediately before administration (T0) and after administration (T1). Psychopathological traits were evaluated using the MMPI-2, including Harris and Lingoes subscales.

**Results:**

The Friedman test revealed significant differences in physiological parameters (χ² = 39.772; df = 5; p < .001). Spearman correlations highlighted specific associations between psychopathological profiles and cardiovascular parameters. At baseline (T0), SBP was inversely correlated with Hysteria (Hy; ρ = -0.886; p = .019) and positively correlated with Paranoia (Pa; ρ = +0.886; p = .019); DBP was negatively associated with Hypochondriasis (Hs; ρ = -0.841; p = .036). After administration (T1), SBP showed negative correlations with Hy (ρ = -0.829; p = .042) and positive correlations with Hypomania (Ma; ρ = +0.829; p = .042), while DBP was positively associated with Psychasthenia (Pt; ρ = +0.886; p = .019). Harris and Lingoes’ subscales further refined these patterns: Hy3 (Fatigue/Malaise) was negatively correlated with HR at T1; Hy5 (Inhibition of Aggression) was negatively associated with HR at both T0 and T1, as well as with SBP at T1. Conversely, Pa1 (Persecutory Ideas), Ma1 (Amorality), and Ma2 (Psychomotor Acceleration) were positively correlated with SBP at T1.

**Conclusions:**

Intranasal esketamine causes an acute and significant increase in blood pressure, without affecting heart rate. However, cardiovascular responses are heterogeneous and modulated by specific psychopathological profiles: anxious-hypochondriacal and inhibitory traits are associated with attenuated responses, while persecutory thoughts, psychasthenia, hypomania and subscales of amoral behaviour/psychomotor acceleration predict an increase in blood pressure. These results suggest the importance of integrating systematic psychopathological assessment into clinical monitoring and personalising therapeutic protocols with esketamine.

**Disclosure of Interest:**

None Declared

